# Development of Car Coating Materials over the Past Decade for Paint Protection Applications—An Overview on the Different Types of Paint Protections

**DOI:** 10.3390/polym17233114

**Published:** 2025-11-24

**Authors:** Umar Nirmal, M. A. Musa, Mohd Yaakob Yuhazri, M. M. H. Megat Ahmad

**Affiliations:** 1 Centre for Advanced Mechanical and Green Technology, Centre of Excellence for Robotics & Sensing Technologies, Faculty of Engineering and Technology, Multimedia University, Jalan Ayer Keroh Lama, Melaka 75450, Malaysia; 2 Faculty of Industrial and Manufacturing Technology and Engineering, Universiti Teknikal Malaysia Melaka, Hang Tuah Jaya, Durian Tunggal, Melaka 76100, Malaysia; yuhazri@utem.edu.my; 3 Department of Mechanical Engineering, Faculty of Engineering, National Defence University of Malaysia, Kem Perdana Sungai Besi, Kuala Lumpur 57000, Malaysia

**Keywords:** automotive coatings, wax coatings, hybrid coatings, surface preparation, sustainable coatings, nanomaterials, future research

## Abstract

In recent years, the automotive industry has experienced increasing demand for advanced paint protection solutions aimed at improving vehicle durability, preserving aesthetic appeal, and promoting environmental sustainability. This paper critically examines the main categories of paint protection coatings on wax, ceramic, graphene, and hybrid formulations by focusing on their chemical composition, application methods, protective performance, and limitations. Wax coatings remain widely adopted due to their affordability and ease of use, though they offer limited longevity. Ceramic coatings, in contrast, provide superior hardness, hydrophobicity, and resistance to scratches, corrosion, and ultraviolet (UV) degradation, albeit with higher costs and complex application procedures. Emerging graphene-based coatings demonstrate exceptional hydrophobicity, thermal stability, and durability, positioning them as potential next-generation solutions, though their environmental and economic feasibility remains under exploration. Hybrid and self-healing coatings further highlight the trend toward multifunctional, intelligent protection systems. This work also emphasizes the critical role of surface preparation in determining coating performance. Future research directions are outlined, including the development of biodegradable, zero-VOC, and intelligent self-aligning coatings, which could significantly advance sustainable automotive surface protection. Overall, this work provides a comprehensive synthesis of current technologies and identifies pathways for innovation in automotive paint protection materials.

## 1. Introduction

Protective coatings play a vital role in extending the durability of automotive surfaces by mitigating environmental degradation and mechanical wear. Automotive coatings are designed to resist UV radiation, acid rain, oxidation, and mechanical damage such as scratches, swirl marks, or chipping. Beyond aesthetic enhancement, coatings serve as a barrier, providing long-term protection against environmental hazards. The shift from labour-intensive manual painting methods to automated coating technologies has significantly advanced the industry over the past century [1]. With the growing emphasis on sustainability and performance, the development of advanced coating systems has become a central focus in both academic research and industrial practice.

The evolution of automotive coating technology reflects continuous advancements in material science and manufacturing. In 1923, nitrocellulose lacquer was introduced as a transparent coating that imparted a glossy finish, though it was highly susceptible to damage from acid rain caused by air pollution [2]. By the 1930s, alkyd enamel paints offered improved durability through molecular bonding within their chemical structure, showing far greater resistance to acid rain compared with lacquers [3]. In the 1940s, the birth of dip-coating technique has provided more uniform distribution and smoother coating finishes, though the process was labour-intensive and not well-suited for large-scale automotive applications. This limitation was addressed in the 1960s with the adoption of electrodeposition (ED) method. This coating technique used electrostatic charging to achieve cost-effective and reliable automated coating. The 1970s saw the introduction of anodic deposition coatings technique that utilized melanized polybutadiene resins as ‘two-layer’ topcoats after the application of base colour coat, producing high gloss surfaces with enhanced weather resistance. This improvement was followed by the incorporation of metallic pigments, such as aluminium flakes, into the original paints that further improved the brilliance and sparkle of automotive finishes [3].

Further advances were achieved in the late 1970s with the “wet-on-wet” technique, combining thinner basecoats with thicker clearcoats to improve durability and lifespan. However, the increased coating weight negatively affected vehicle fuel efficiency, a concern for automotive manufacturers [4]. To address this issue, water-based basecoats were adopted in the 1980s. This technique enabled high throughput and uniform film thickness while reducing environmental impact. By 2002, over 70% of automotive coatings were waterborne, reflecting the industry’s transition to environmentally compliant and cost-effective solutions [1,5]. This shift was further reinforced by standardizing the adoption of inorganic pretreatments, cathodic electrodeposition, and powder coating methods that aligned with global environmental regulations [6,7].

At present, automotive detailing industries apply secondary protective coatings to preserve vehicle exteriors against UV exposure, acid rain, bird droppings, chemical contaminants, abrasion, swirl marks, and scratches. These coatings also protect against primer oxidation, which results from prolonged sunlight exposure. In recent years, “smart coatings” have emerged as a promising coating, offering not only surface protection but also advanced functionalities such as self-healing, super hydrophobicity, self-stratification, self-sensing, and high durability under severe weather conditions [8,9,10,11].

**Figure 1:**
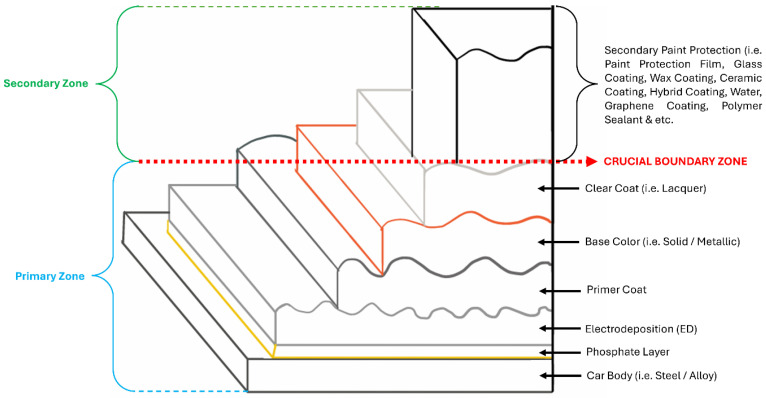
Different types of paint protection coatings on a typical passenger car vehicle.

As illustrated in Figure 1, automotive coatings can be classified into two main zones: the primary zone, applied during vehicle manufacturing, and the secondary zone, applied after purchase by car owners or professional detailers [12]. The boundary between these zones is critical, as the quality of the secondary protective coating largely depends on the condition of the underlying primary coating. Consequently, proper surface preparation is essential before secondary applications are performed. This review critically examines recent progress in secondary automotive coatings, with a focus on wax, ceramic, and advanced alternatives, while also emphasizing the importance of surface preparation as a determining factor in coating performance.

**Figure 2:**
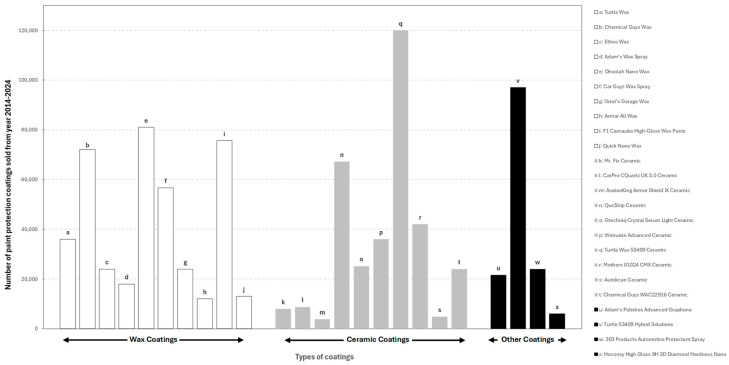
Various types of paint protection coatings sold from year 2014 to 2024. **Source:** Online shopping platforms from Amazon URL (accessed on 11 January 2025): https://www.amazon.co.jp/, eBay URL (accessed on 11 January 2025): https://www.ebay.com/, Shopee URL (accessed on 11 January 2025): https://shopee.com.my/&Lazada URL (accessed on 11 January 2025): https://www.lazada.com.my/ **Keywords used:** Wax, ceramic, and other car coatings.

**Figure 3:**
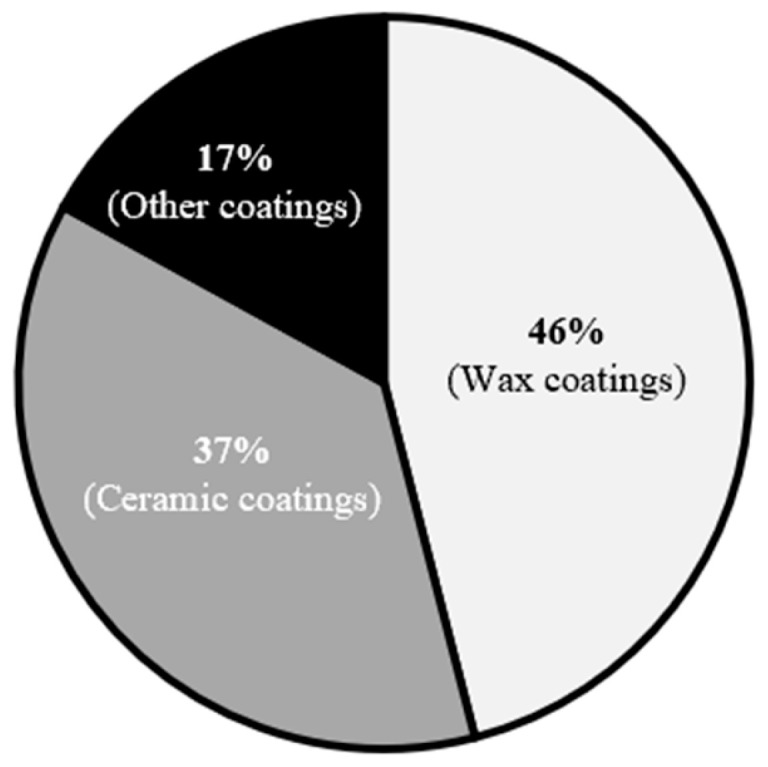
Preferred type of paint protection by consumers from 2014 to 2024. **Note:** Paint protection is only taken for application of passenger car vehicles.

It is to be noted that the secondary zone, as illustrated in Figure 1, is intended to maintain and extend the durability of the primary coating originally applied by automotive manufacturers. Consequently, the secondary zone encompasses a wide range of products used by professional car detailers and increasingly by end-users. To gauge consumer preferences for secondary paint-protection products, we surveyed product listings and buyer reviews on four major online marketplaces (e.g., Amazon, eBay, Shopee, and Lazada) for the period 2014–2024. Figure 2 summarizes the range of products available across those platforms: items labelled ‘a’–‘j’ correspond to wax-based products, ‘k’–‘t’ to ceramic-type coatings, and ‘u’–‘x’ to other protection products (for example, graphene-infused, hybrid, diamond-like coatings, and protection sprays). From this inventory, we extracted aggregate purchase/preference metrics, which are presented in Figure 3. The extraction and classification were performed with a focus on products intended for passenger cars only, and listings were screened manually for relevance and reviewer credibility.

Figure 3 shows that, for the period examined, wax-type products account for approximately 46% of consumer purchases, while ceramic-type coatings represent about 38%, and other specialty coatings comprise the remaining ~17%. Several factors help explain this distribution. Wax products are inexpensive and easy to apply (e.g., many are in the form of “spray-and-wipe type ”), require a short setting time, and are therefore well suited to ‘Do It Yourself’ (DIY) users; these characteristics make waxes popular among non-specialist car owners. By contrast, ceramic, glass, and other high-performance coatings typically demand thorough surface preparation and professional application to achieve the advertised durability and appearance. High-hardness coatings (e.g., silica-rich “glass” coatings) are effectively semi-permanent: once applied, they can lock in pre-existing defects (tar, swirl marks, scratches, and dust) because their hardness and chemical bonding limit post-application correction. These practical and economic trade-offs help explain the relative popularity of lower-cost, user-friendly waxes despite their shorter service life.

**Figure 4:**
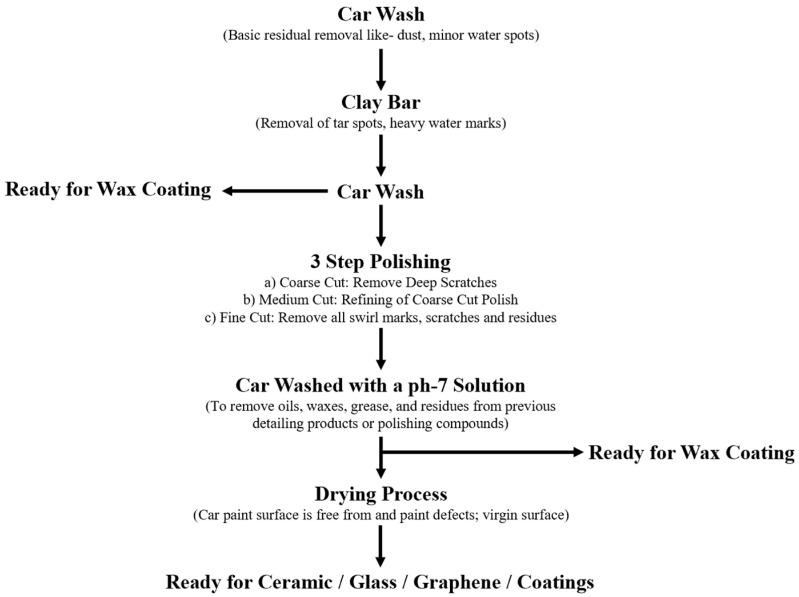
Surface preparation on a car exterior body before undergoing paint protective coatings. **Note:** Arrows indicates the flow of works upon the completion of previous work.

Prior to applying any secondary coating, surface preparation is critical to ensure proper adhesion and long service life. Typical preparatory steps include a complete wash, clay-bar decontamination, degreasing, paint correction (polishing) to remove swirls and light scratches, and a final wipe-down before coating application. Figure 4 illustrates a representative surface-preparation workflow for secondary coatings. Many coatings (including most ceramic and graphene formulations) also require strictly clean, defect-free substrates to form the intended chemical or mechanical bond with the clear coat; inadequate preparation therefore shortens effective coating lifetime and can lead to premature failure or aesthetic defects. When properly applied, however, these coatings exhibit desirable properties such as hydrophobicity, UV resistance, and enhanced protection against contaminants and minor mechanical damage [13].

Prior to any type of paint protection, surface preparation is crucial to enable the application of coating bonds well with the base coat/material. In this section, we will illustrate the crucial steps taken for surface preparation prior to the secondary zone coating; refer Figure 1. It is to be reminded here again that due to the broad scope of paint protection coatings, this review paper will focus only on the different types of coatings used at the secondary zone of the car exterior body. Hence, the assumption is that the exterior car paint is close to perfect (i.e., minor to moderate paint damage) after the car is sold to the buyer. The buyer than perform an enhancement of the paint protection as per illustrated in the secondary zone. To get a glimpse of the crucial stages of surface preparation before the application on the types of paint protection available in the secondary zone, Figure 4 is presented. From the figure, it is to be noted that surface preparation is crucial prior to paint protective coating because it can enhance the durability of the coating to different applications. By comparison, most types of coatings have good hydrophobic properties which repel water marks on the car surface. They also exhibit good UV resistance, prevention of swirls, minor to major scratches and resistance to chemical contaminants caused by acid rain, bird dropping or extreme weather condition [13]. However, they differ mainly on the longevity on retaining its protection to the car exterior paint. This is due to the surface preparation conducted by the user or a detailer before applying any types of paint protection coatings. In other words, if the surface preparation is poor, i.e., evidence of dirt or fine debris trapped within paint scratches, then the coating may not last be based on its specified time. This is due to the dirt, and debris may get loose due to vibration of the surface which will remove the coating applied on it.

In the remainder of this review, we focus on secondary paint-protection systems applied after vehicle delivery, under the assumption that the factory (primary) coating is largely intact (i.e., only minor to moderate paint defects are present). We limit the scope to liquid- or film-applied coatings—notably waxes, ceramic/glass (silica-based) coatings, and emerging graphene- and hybrid-type chemistries—and do not consider Paint Protection Films (PPF), which are distinct, preformed sheet products rather than applied coatings.

Before diving into the different types of coatings, it is crucial to understand the primary objectives of a coating in terms of its hardness and water beading properties of a coated surface [14]. Different coatings exhibit varying levels of hardness. Coating hardness is an important factor since its primary function is to protect the surface from any type of mechanical wear such as pitting, scratches and swirl marks. In other words, the higher the hardness value of a coating, the higher the resistance of the surface subjected to mechanical wear. Paint protective coatings also offer great protection against chemical and erosion wear. This is primarily due to the chemical composition of the coatings that causes water to bead. The underlying principle involves the contact angle of the water droplets with respect to the coated surface. Typically, a coating that results in a contact angle of more than 90° will cause the water to bead on the coated surface. This phenomenon is also known as hydrophobicity, i.e., low surface wettability, which plays a crucial role in preventing chemical and erosion wear, facilitating self-cleaning effects and preventing water marks.

**Figure 5:**
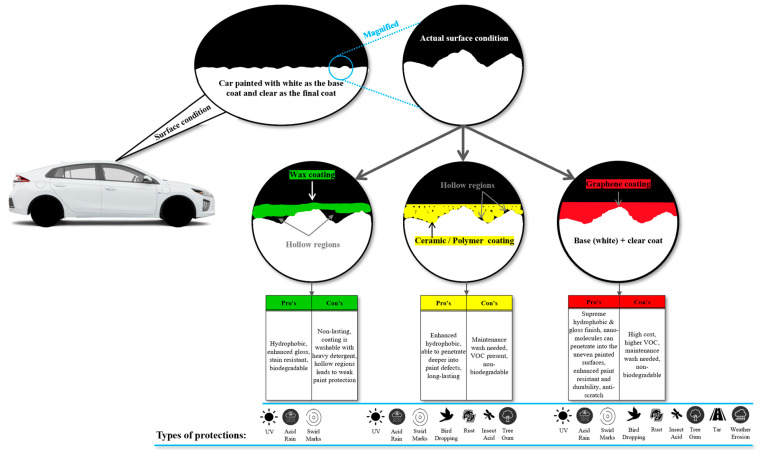
The different types of coatings, their applications and the types of paint protection offered with respect to the original car paint.

Figure 5 illustrates the schematic illustration of the different types of coating used as paint protection on passenger car vehicles. From the figure, it is understood that the surface finishing of the car’s paint from the manufacturer is never perfect; i.e., there are minor scratches that are not visible to the naked eye. These scratches accumulate dirt and debris over time, making the paint look dull and worn out. To overcome this, different types of protective coats are introduced, such as wax, ceramic, polymer, and graphene. Specifically, wax paint protection is a blend of carnauba wax with other chemical substances to serve the purpose of coating the entire car surface in general. However, it must be noted that the molecules of wax-type coatings are relatively large, thereby limiting the wax coating from entering deep scratches on the painted surfaces. This creates hollow regions, which are the crucial weak points of the wax coating. These weak points soon rupture through the top of the wax coating due to vibration and harsh wind conditions, causing the coating to disintegrate. Eventually, the coating will be removed as patches, which becomes quite obvious when there are un-shining spots on the car, indicating the absence of the wax coating. On the other hand, wax coatings are prone to dilution with heavy rain, where the catalyst would be the acid contained in the rain. This accelerates the removal of the wax coating over time, and the painted surface will be once again exposed to the harsh environment. This will damage the paint surface due to UV light, and swirl marks caused by improper car washing, bird droppings, tar spots, and other factors are presented in Figure 5.

Moving on to ceramic or polymer-based coatings, they contain nano-molecules, which allow the coating to enter into the uneven painted surface. However, penetrating into the deep scratches remains a challenge for this type of coating due to the size of the molecules. Hence, there is still evidence of hollow regions, but to a minimum extent as compared to wax coating. Since this type of coating is widely accepted among most car detailers, it is due to the fact that it provides enormous protection to the car’s painted surfaces. The final appearance after the application of this type of coating results in a smooth and shiny look on the car’s exterior. It has high resistance to disintegration during harsh driving conditions, but the main failure of the coating will arise from the hollow regions. Some of the protection it offers include excellent resistance to UV light, acid rain, swirl marks, bird droppings, rust, insect acid, and tree gum/sap. Some of the pros and cons are also tabulated in Figure 5, respectively.

Lastly, when we examine graphene-based coatings, they are often regarded as the most superior option compared to other types of coatings. This is primarily due to the chemical structure of graphene, which is exceptionally small, smaller than many nanoscale particles and able to penetrate deeply into paint defects on a car’s surface. In addition, the adhesion between the coating and the car’s paint is significantly stronger than that of other coatings, as a primer is typically applied before the actual graphene coating is carried out. The final result is a remarkably smooth, silky finish that repels water effectively and withstands harsh environmental conditions, as illustrated in Figure 5. Although this type of coating provides excellent protection for automotive paint, it is relatively expensive due to a labour-intensive application process and the high cost of the material itself. To maintain the durability of this coating, detailers commonly recommend periodic maintenance washes, during which the vehicle undergoes a thorough coating inspection under an array of lighting arrangement. This helps to identify areas where the coating has overlapped or degraded over time, allowing necessary corrections to be made using state-of-the-art chemicals which, however, may pose environmental concerns.

## 2. Introduction to ‘Wax’ Coating

In the automotive industry, wax-type coatings are widely used to protect and enhance the appearance of painted surfaces. These coatings form a hydrophobic barrier that repels water and helps prevent contaminants from adhering to the paint. Among natural waxes, carnauba wax—derived from palm leaves—is the most common ingredient in automotive waxes due to its hardness, high melting point, and ability to impart a deep, glossy finish [15]. Chemically, carnauba wax is a complex mixture rather than a single compound. It is primarily composed of aliphatic esters, with myricyl cerotate (C_56_H_112_O_2_) as a major component, along with free fatty acids, fatty alcohols, hydrocarbons, and resins. The composition varies depending on the source and the processing method [16].

**Figure 6:**
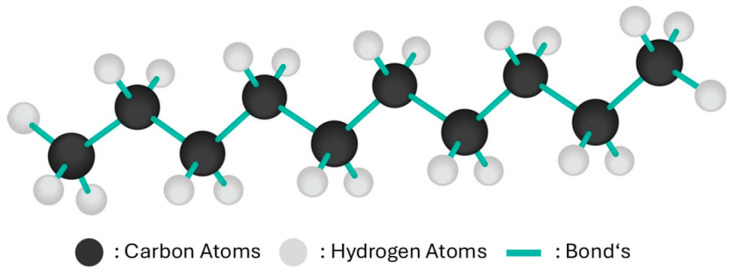
Chemical structure paraffin wax also known as ‘Decane’’ C_10_H_22_.

Although carnauba wax exhibits excellent hardness and water resistance, it can be brittle in its pure form, which makes it difficult to achieve uniform application over large surfaces. To improve flexibility and ease of application, it is often blended with softer natural waxes, such as beeswax, or with synthetic additives [15]. Figure 6 [16] illustrates the molecular structure of paraffin wax (decane, C_10_H_22_) as a representative hydrocarbon chain used in some wax formulations. Modern wax-based coatings increasingly combine natural waxes with synthetic polymers, silicones, or polytetrafluoroethylene (PTFE) to improve durability, UV resistance, and scratch protection [16].

Modern automotive wax formulations often incorporate synthetic waxes and polymers such as polytetrafluoroethylene (PTFE), silicones, and other performance-enhancing additives. These may be combined with or substituted for natural waxes to increase durability, weather resistance, and chemical stability. Such synthetic blends have been shown to provide superior protection against UV radiation, environmental pollutants, and minor to moderate surface scratches compared with natural wax alone [16]. By modifying both the chemical composition and physical film properties, manufacturers have developed wax products that better meet consumer demands for longevity and protection while maintaining ease of application. The primary protective mechanism of wax coatings lies in their hydrophobicity. By lowering the surface energy of the paint, waxes reduce the adhesion of water and contaminants. This property is commonly assessed by water contact-angle measurements, where higher angles indicate greater hydrophobicity [16]. Superhydrophobic surfaces are defined by contact angles greater than 150°, usually enabled by micro- and nanostructured topographies. While superhydrophobic coatings are relevant, specific contact angle values for automotive waxes like carnauba wax are not readily available and it depends on the specific formulation and the substrate surface [17,18,19,20,21]. Although conventional automotive waxes do not achieve such extreme values, they provide sufficient water repellency for short-term protection. Placido et al. [22] reported that commercial wax coatings show initial contact angles between 71.17° and 94.85°. After one week, the contact angle values remained largely unchanged, suggesting stable short-term hydrophobicity but limited long-term durability. Long-term studies, however, remain limited.

Automotive waxes are available in three principal forms: paste, liquid, and spray. Paste waxes are traditionally packaged in tins and contain the highest proportion of carnauba wax. They are labor-intensive to apply and buff but deliver superior gloss and depth of shine [22]. Under typical conditions, paste waxes last for two to four months. On the other hand, liquid waxes are easier to spread and can be applied by hand or polishing machine, which make them ideal to be used by beginners. They often contain a blend of carnauba, polymers, and resins, offering a balance between durability and user convenience, with protective lifetimes extending up to one year depending on formulation [23]. Spray waxes provide the greatest ease of application but the shortest durability, typically two to four weeks. They are primarily marketed as maintenance boosters to refresh gloss and hydrophobicity between applications of more durable products.

The protective effectiveness of wax coating is influenced by multiple factors including wax type, formulation, application technique, washing frequency, and environmental exposure. Waxes are susceptible to erosion from detergents, rain, and UV degradation, and thus require frequent reapplication to maintain performance. Manufacturers often blend natural and synthetic components to enhance durability while preserving desirable visual effects [23]. According to Poozesh et al. [24], the compatibility of wax formulations with different automotive paint systems (solid, metallic, or clear-coated) is critical to achieving reliable adhesion and optimum gloss. Anthony [25] similarly emphasized that waxes can enhance surface appearance and water repellency, provided that they are matched appropriately to the substrate.

### Review on the Different Types of Wax Coatings by Brands

There are various types of wax coatings for car paint protection produced by leading manufacturers worldwide. In this review, we compiled a selection of the most prominent wax products currently in use, together with their key characteristics and typical paint-protection lifetimes after a single application. These details are presented in Table 1. It should be noted that Table 1 does not represent a ranking from “most preferred” to “least preferred”; rather, it lists products most frequently recommended by professional detailers and car owners. Moreover, because many users lack prior detailing experience, ease of application may also contribute to the popularity of these wax products.

## 3. Introduction to ‘Ceramic’ Coating

Ceramic coatings, also referred to as glass, silica, or nano coatings, represent a significant advancement in automotive paint protection. Their formulations generally consist of three key components: resins, solvents, and additives [33]. In the pursuit of the so-called ‘optimum paint protection’ through ceramic coatings, numerous chemical industries are competing to develop formulations that combine high hardness with superior protective performance [34,35]. The protective performance of ceramic coatings is primarily attributed to their high silica (SiO_2_) content, often supplemented with titanium dioxide (TiO_2_) to enhance hardness and ultraviolet (UV) resistance [21,36]. Unlike waxes or polymer sealants that rest on the paint surface, ceramic coatings form chemical bonds with the clear coat, producing a durable and semi-permanent protective layer. During application, hydrolyzed SiO_2_ precursors react with surface hydroxyl (-OH) groups to form Si–O–Si covalent bonds, which penetrate micro-pores in the paint. The resulting three-dimensional crosslinked lattice cures within 24–48 h, often accelerated by infrared (IR) light, producing a hard, glossy, and chemically resistant layer with hardness ratings exceeding 9H [37]. Once cured, ceramic coatings provide superior hydrophobic and oleophobic properties, high gloss, and excellent resistance against UV radiation, surface scratches, acid rain, bird droppings, tree sap, snow, and ice [38,39]. Their chemical bonding and high hardness distinguish them from conventional waxes, enabling long-term durability that can extend from one to several years depending on formulation and maintenance.

**Figure 7:**
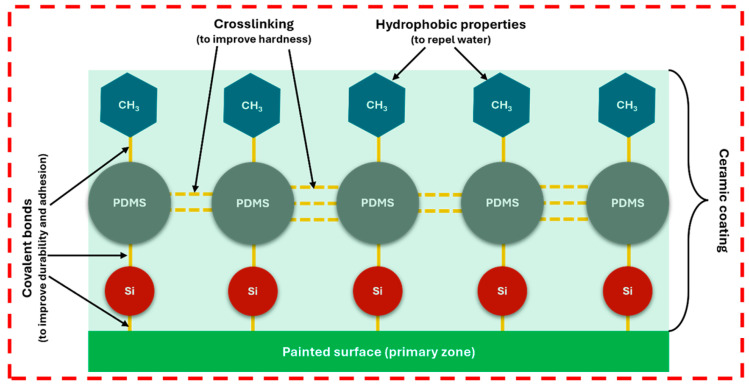
Chemical structure of a typical ceramic coating and its important bonds. **Note:** Red dotted line represents controlled environment govern by temperature, humidity & air quality.

In addition to SiO_2_, silicone-based polymers such as polydimethylsiloxane (PDMS) are often incorporated as resins in ceramic coating formulations [40]. Although PDMS provides flexibility and water repellency, it has poor adhesion to painted surfaces when used alone. To overcome this limitation, PDMS is chemically modified with silane coupling agents that promote covalent crosslinking between polymer chains and the substrate, enhancing its hardness up to 10H and above [41]. Methyl (-CH_3_) groups within PDMS play a critical role in imparting hydrophobicity, while silane crosslinks improve adhesion and mechanical stability. A representative chemical structure of ceramic coating linkages is illustrated in Figure 7.

It is to be reminded here that the entire application of ceramic coating to the painted surface needs to be carried out in a controlled room with correct room temperature, humidity and air quality [42,43]. For instance, the recommended room temperature by most car detailers is around 15 to 27 °C [44]. If the temperature is below 15 °C, the coating applied will slow down the solvent evaporation and curing process which can lead to a soft, incomplete or a weak cure. Consequently, this reduces the longevity of the coating. When the coating is performed at temperatures above 27°, the solvents of the coating will evaporate very fast making it difficult to level or “wipe off” the applied coating. This will cause streaking, hazing, or premature curing before a stable covalent bond forms. Humidity plays a crucial role in the application of coatings. Environments with high humidity can directly interfere with the formation of stable cross-links and covalent bonds. This will result in poor ‘dull looking’ coating or a premature cure is formed on the painted surfaces. On the other hand, when the humidity is too low, the applied coating will flash dry quickly during application making it difficult to buff off the coated surfaces which often leads to an uneven coat [45]. Lastly, the challenge of coating application is that it should be performed in a ‘clean room’ to ensure the coating bonds directly to the prepared surface. This is crucial as it will result in a smooth and glass-like finish surface. If the environment is polluted with dust and debris due to the polishing residue, this can interfere with the formation of the covalent and cross-links of the coating, which will result in an uneven coating containing foreign particles. These particles will give an overall impact to the aesthetic quality of the final coating [46]. In a nutshell, the application of ceramic coating is critically influenced by the environment as mentioned above. This concept is also schematically presented in Figure 7, where the red dotted line indicates a controlled environment.

Because ceramic coatings cure via hydrolysis and condensation, they may release methanol or other alcohols as by-products, classified as Volatile Organic Compounds (VOCs) [40]. Elevated VOC emissions present both health and environmental hazards, highlighting the challenge of formulating long-lasting coatings with reduced VOC content [47].

Application of ceramic coatings requires meticulous surface preparation, including washing, clay-bar decontamination, degreasing, and polishing to remove visible defects such as swirls, scratches, or tar spots. The coating is then applied in small sections, typically in shaded environments to avoid premature curing. Moisture in the air initiates hydrolysis, and curing can be accelerated using infrared (IR) lamps. A final buffing step with microfiber cloth ensures an even, glossy finish. Depending on formulation and environmental exposure, ceramic coatings typically provide effective protection for one to three years, although periodic maintenance washes are recommended to maximize performance.

Numerous studies have highlighted the versatility of ceramic coatings across industrial applications. Köse et al. [48] demonstrated their superior wear, corrosion, and thermal resistance, making them suitable for components exposed to extreme conditions. Aimovi-Pavlovi et al. [49] emphasized the importance of additives and fillers in improving coating adhesion and stability, particularly in casting applications. Xuebai et al. [50] developed a cost-effective Al_2_O_3_-stearic acid/polyurethane hybrid ceramic coating via spray application, achieving super hydrophobicity and enhanced corrosion resistance on automotive steel substrates. These studies illustrate how tailoring ceramic formulations with additives or hybrid structures can significantly expand their protective functions beyond conventional automotive use.

To evaluate surface interactions, ceramic coatings are commonly categorized by their water contact angle. Superhydrophobic surfaces exhibit contact angles above 150°, hydrophobic surfaces range from 90–150°, while untreated or degraded surfaces display hydrophilic (<90°) or superhydrophilic (<10°) behavior [51]. Table 2 summarizes these classifications, illustrating the effect of coating quality on water repellency.

### Review on the Different Types of Ceramic Coatings by Brands

A variety of commercial ceramic coatings are available globally, differing in chemical composition, ease of application, and durability. Table 3 summarizes representative products, their characteristics, and expected protection lifetimes. The listed products are not ranked based on the ‘most preferred’ and the ‘least preferred’, but rather represent popular choices as frequently recommended by both professional detailers and consumers.

## 4. Other Types of Coating

***(i)***  ***Polymer coatings/Synthetic sealants***

Polymer-based coatings, also referred to as synthetic sealants, are formulated from long-chain synthetic polymers that crosslink upon application to painted surfaces [59]. Typically derived from petroleum-based feedstocks, they are not biodegradable and thus raise environmental concerns. Polymer sealants are available in spray or paste forms and generally provide protection for 4–6 months. Their benefits include a deep, reflective finish, resistance to environmental contaminants, and the ability to mask fine scratches and swirl marks. Application involves spreading the sealant, allowing 10–15 min of curing, and buffing with a microfiber cloth.

**Figure 8:**
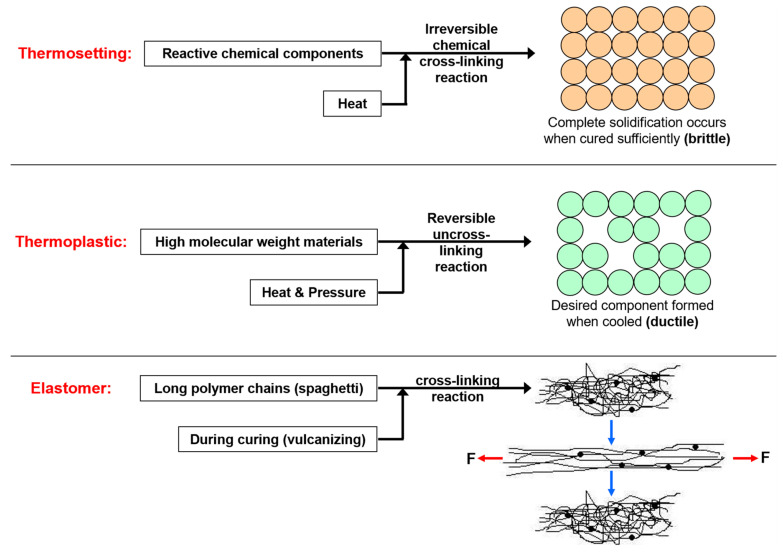
Schematic illustration of the different types of polymers. **Note:** Reprinted with permission from Ref. [65]. Copyright © 2014, Elsevier.

Polymers can be broadly classified as thermosets, thermoplastics, and elastomers [60]. Thermosets, such as polyester-based coatings, form irreversible crosslinked networks that yield a hard, durable protective layer, and are therefore widely employed in automotive applications. Thermoplastics, with their reversible bonding, are increasingly used in the development of self-healing coatings [61], while elastomers are mainly applied in PPF [62]. Figure 8 summarizes the different types of polymers according to their category. In addition, corrosion-inhibiting pigments such as zinc or aluminum phosphates are often incorporated into polymer coatings to improve resistance against chemical degradation [63]. Despite their moderate durability and affordability, polymer coatings are associated with high VOC content, which limits their environmental compatibility [64].

***(ii)***  ***Graphene coatings***

Graphene-based coatings have recently emerged as high-performance alternatives to ceramic coatings. Graphene, a two-dimensional allotrope of carbon arranged in a honeycomb lattice, exhibits exceptional mechanical strength, thermal stability, and barrier properties [66,67,68]. When formulated into coatings, graphene and its derivatives graphene oxide (GO) and reduced graphene oxide (rGO) provide enhanced hydrophobicity, chemical resistance, and thermal stability compared to conventional SiO_2_-based coatings. The oxygen functional groups present in GO (hydroxyl, epoxide, carbonyl, and carboxyl) can limit stability, but partial reduction to rGO improves conductivity, hydrophobicity, and chemical durability [69].

**Figure 9:**
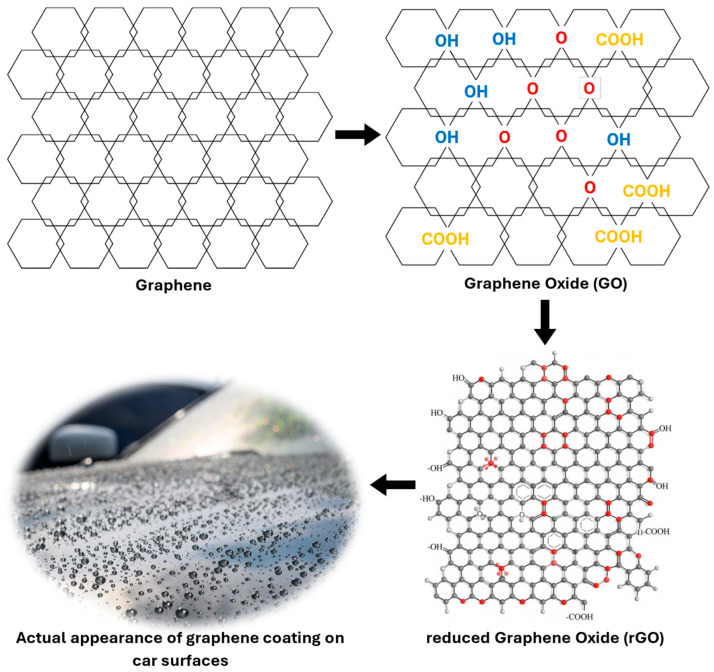
Chemical structure of graphene, GO and rGO and its actual application for coating painted car surfaces [71]. **Note:** COOH—carboxyl group; HO or OH—hydroxide ion; O—oxide.

In automotive applications, graphene coatings are typically blended with silica or polymeric binders to produce a thin, glossy, and highly hydrophobic protective layer. Compared with ceramic coatings, they exhibit greater flexibility, higher contact angles, and improved resistance to cracking or chipping. Reported service lifetimes range from 5 to 10 years depending on formulation and application quality. Application procedures are similar to ceramic coatings, requiring thorough surface preparation and careful curing. Figure 9 illustrates the chemical structures of graphene, GO, and rGO.

In a recent study reported by Bui and co-researchers [70] on the effect of graphene addition in inorganic polymer paint to enhance its physical and chemical properties, it was concluded that a 0.05% graphene concentration increased the paint’s heat resistance by 5%, while corrosion resistance improved by 20% compared to the control sample (i.e., the painted sample without graphene). Remarkably, Scanning Electron Microscopy (SEM) imaging showed that graphene addition resulted in a smoother surface morphology. There was evidence of improved coating adhesion and a noticeable reduction in particle porosity, leading to better mixture uniformity. However, it was also revealed that adding higher amounts of graphene worsened the coating performance due to poor dispersion and the formation of surface defects at the graphene–paint interface. In summary, the study highlights that an optimal graphene concentration is required for effective paint protection, which can be determined through experimental testing following standardized procedures such as the heat resistance test, pencil hardness test, erosion test, wear tests and water absorption test.

### Review on the Different Types of Graphene Coatings by Brands

A wide range of graphene-based coatings are now commercially available, often marketed as hybrid graphene–ceramic formulations [72]. Table 4 summarizes representative products, their primary characteristics, and expected protection lifetimes. The listed products reflect popular choices among both professional detailers and consumers but are not presented in any ranking order.

## 5. Summary of Reviewed Works

This review highlights several crucial insights regarding the current state and practical challenges of automotive paint protection coatings. These points encompass cost considerations, consumer preferences, industry practices, and technical factors that collectively influence the performance, accessibility, and reliability of coating technologies. The key findings are summarized as follows:

- The cost of paint protection coatings generally follows the order: wax < ceramic << graphene, with wax being the most affordable option and graphene the most expensive.
- Spray-type coatings are generally preferred by car owners due to their ease of use and suitability for DIY application after routine washing. In contrast, liquid-type coatings are typically applied by professional detailers, offering significantly greater durability but at a higher cost. The elevated expense reflects the labour-intensive surface correction required prior to application, a process that may take one to three days depending on weather conditions and curing time.
- Many car owners are aware of the different types of paint protection available. Literature suggests that new car owners, in particular, are more willing to invest in durable and long-lasting coatings, as their vehicles typically exhibit minimal or no surface defects compared to used cars without prior paint protection. Consequently, the overall cost of application is relatively lower, since only limited paint correction work is required by professional detailers.
- The pursuit of an “optimum” coating—one that offers maximum resistance to environmental and mechanical defects—has fuelled intense competition among manufacturers. However, a persistent challenge lies in formulating coatings that are both high-performing and environmentally sustainable. When reducing VOC emissions remains critical: while low-VOC formulations are safer for users and less harmful to the environment, they often compromise coating quality and durability. Consequently, manufacturers must balance performance with regulatory compliance, as coatings are subject to strict legislative standards before being released to the market.
- In some cases, coatings are misleadingly marketed to maximize profit—for example, products advertised as ceramic coatings may in fact be wax-based. Such misrepresentation often results in buyers paying two or more times the actual value of the product. To avoid these pitfalls, consumers are strongly advised to purchase coatings from reputable brands, certified detailers, or trusted retail outlets.
- Some coatings lack a valid brand or legitimate manufacturer and are often generically labelled, for example, as “glass-ceramic coatings.” When such unverified products are purchased online or off-the-shelf and applied to vehicle surfaces, they may contain harmful chemicals that degrade the clear coat, progressively thinning it with repeated use. This leaves the underlying paint vulnerable to environmental exposure, increasing the risk of cracks, chips, and eventually rust formation.
- In some cases, car detailers prioritize profit over quality, leading to inadequate surface decontamination and preparation prior to coating application. Consequently, defects such as tar spots, scratches, and swirl marks often remain visible beneath the applied layer. While specialized chemicals can be used to strip the coating and correct these flaws, large-scale or repeated use may negatively interact with advanced coatings such as ceramic, glass, or graphene resulting in smearing and an uneven finish that diminishes gloss and visual appeal under light.
- It should be noted that coating longevity is strongly influenced by hardness, which is a key determinant of durability. As hardness increases, the coating becomes increasingly difficult and sometimes nearly impossible to remove, while the use of chemical removers may further compromise surface quality. This underscores the importance of proper surface preparation prior to application. Notably, two detailers using the same brand of coating may deliver very different outcomes depending on the rigor of their preparation process, even if their service costs vary. Ultimately, inadequate surface preparation undermines the protective performance of the coating and diminishes the final aesthetic appearance of the vehicle under visible light.

## 6. Future Research

Following this extensive review, several potential avenues for future research in the development of car coating materials were identified. These directions merit further investigation and are outlined as follows:

- Recent studies indicate that combining ceramic coatings with other chemical components to produce hybrid formulations can significantly enhance coating longevity while imparting superhydrophobic properties. Numerous investigations on hybrid coatings have been reported, and continued research is essential to establish an optimal chemical blend that balances performance with user safety and environmental sustainability. In this regard, the development of biodegradable coatings derived from natural plant- and animal-based sources is proposed as a promising direction. However, the challenge remains unaddressed since according to the literature, it is noted that lowering the VOC content of a coating can reduce the longevity of paint protection. Hence, more research is proposed in this area to get a correct formulation of biodegradable hybrid coatings with a justified amount of VOC values for optimum paint protection.
- Most car coatings contain a measurable number of VOCs. Although these coatings generally comply with national regulatory standards, future research should prioritize the development of zero-VOC formulations to minimize their environmental impact and improve user safety.
- Intelligent coatings represent a promising future direction for automotive paint protection. Although this concept remains largely unexplored, the central idea is to enable coatings to self-align at the atomic or molecular level, thereby forming a uniform layer of consistent thickness regardless of the application method. Such a feature would overcome one of the key limitations of current practices, where manual application through rubbing or spreading often results in overlapping layers and non-uniform coating thickness after solidification.
- Spray-type coatings have been widely adopted and have demonstrated excellent water-repellent properties. However, whether applied manually or robotically, the spraying process often produces overlapping layers, leading to uneven coating thickness. This not only compromises coating uniformity but may also add unnecessary weight to the vehicle, with potential implications for fuel efficiency. Consequently, the development of intelligent coatings capable of achieving self-regulated, uniform deposition remains an unresolved research challenge.
- Self-healing coatings have been reported to restore minor damages such as light scratches and swirl marks. However, a major limitation is their dependence on external stimuli—typically heat—to activate the healing process. This requirement restricts their effectiveness in colder regions where sunlight and UV exposure are limited. To address this, the concept of an “e-self-healing” coating has been proposed, wherein a mild electrical current induces molecular realignment, allowing the coating to recover its original properties. This mechanism relies on the ability of conductive components within the coating to reorganize under electrical stimulation. Nevertheless, further research is required, as such coatings are commonly formulated with conductive materials—such as iron (Fe)—which may increase the overall weight of the vehicle.

By addressing the above points, next-generation automotive coatings can evolve beyond simple protective barriers to multifunctional, sustainable systems that ensure long-term durability, enhanced aesthetics, and improved environmental compatibility.

## Figures and Tables

**Table 1:** Different types of wax coating products offered by different manufacturers.

Product	Product Characteristics	Manufacturer	Average Paint Protection Time
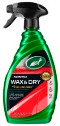 Wax & Dry Spray Car Wax	Two in one, wash and wax spray type. Advanced drying agents repel water from wet surfaces to cut back on drying time. Minute Wax Shine technology adds a layer of instant shine and protection. Product contains Acetaldehyde and Ethylene Glycol which can cause cancer and birth defects [26].	Tuttle Wax	2–4 weeks
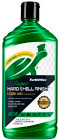 Super Hard Shell Liquid Car Wax	Shake before use. Comes in liquid form and provides protection from light scratches and swirls. Safe on most paint surfaces. Application by microfiber cloth via apply and buff. Buffing is done after 1 min of waiting time after coating. Contains Acrylamide which can cause cancer and birth defects [27].	Tuttle Wax	48 weeks
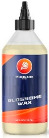 Glossome Wax	Shake before use and surface preparation should be free from any defects. Super-long-lasting synthetic sealer. Long lasting hydrophobic deep gloss. Mixture of Teflon, resins, polymers, and synthetic and organic waxes. Provides hyper-water beading and protection. Application is by applying, wait 5 min before buffing. Protects the color and the brightness of the vehicle’s paint with UV protection. Product may cause eye irritation, respiratory irritation and gastrointestinal irritation if wrongly used [28].	Phoenix E.O.D. where E.O.D stands for ‘Evolve or Die’	36 weeks
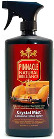 Pinnacle Crystal Mist Detail Spray	Formulated with restructured gloss-enhancing crystalline polymers, nourishing oils, and real Brazilian carnauba wax. Provide instant burst of shine and slickness. Crystal Mist lubricates the paint surface to remove light contamination such as this, without scratching or marring the finish. Paint will appear clean, vibrant and brilliantly glossy. Meets all Volatile Organic Compound (VOC) standards. Best used on black and red paints [29].	Pinnaclewax	1–2 weeks
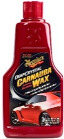 Meguiar’s Deep Crystal Carnauba Wax	High quality carnauba wax for long-lasting protection to clear coat and single stage paint. Creates brilliant look shine and locks it in with an extremely durable wax barrier. Offers protection against effects by the sunlight. Recommended product after 3 stages polishing namely to seal in rich gloss with brilliance and create a protective coating. Application is by applying, wait 2–3 min before buffing [30].	Meguiar’s	4 weeks
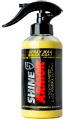 Spray Wax Quick Coat	Application is by spray on and wipe off. Contains Brazilian Carnauba for deep gloss finish look. Leaves no streaks and no residue. Protection against UV Rays and environmental contaminants. Safe for all car surfaces. Regular use is recommended for continuous paint protection [31].	Shine Armor	6 weeks
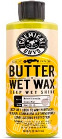 Butter Wet Wax	Formulated from a blend of natural Carnauba, polymers and resins. Can be used on top of ceramic coating for an extra layer of shine and protection. Not recommended for matte finishes. Resistance against UVA and UVB rays. Application method is by wipe on-wipe off method. Can be applied on wet or dry surface and under direct sunlight [32].	Chemical Guys	3–4 weeks

**Table 2:** Schematic view of water droplets in different contact angles with respect to the painted surface conditions [51].

Schematic View	Surface Treatment	Contact Angle to Surface	Characteristics	Range of Hydrophilicity	Range of Hydrophobicity
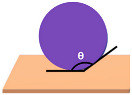	Surface treated with high end ceramic coating (Ex: PDMS–Si–CH_3_)	150^o^ < θ < 180^o^	Superhydrophobic (i.e., highly water repellent, form full water droplets that beads from the surface)	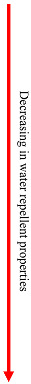	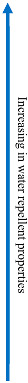
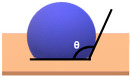	Surface treated with normal ceramic coating (Ex: Si-O-Si)	90^o^ < θ < 150^o^	Hydrophobic (i.e., average water repellent, form ¾ water droplets that beads from the surface, leaving minimum water marks on the surface)		
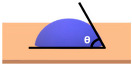	Surface has no any sort of coating	10^o^ < θ < 90^o^	Hydrophilic (i.e., average water attraction, little or no beading from the surface leaving water marks)		
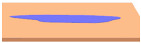	Surface has no any sort of coating	0^o^ < θ < 10^o^	Superhydrophilic (i.e., highly attracted to water, 100% in contact with surface and does not bead)		
Note: 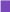 —Blue colour represents water; 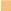 —Beige colour represents painted surface with a given condition; treated/untreated					

**Table 3:** Different types of ceramic coatings offered by different manufacturers.

Product	Product Characteristics	Manufacturer	Average Paint Protection Time
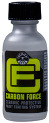 Carbon Force Ceramic Coating	Offers DIY detailers professional-grade protection with its liquid formula and easy application. Using ceramic nanotechnology, it creates a durable, bonded layer that shields against environmental contaminants, UV rays, and wear and tear for up to 5 years. The coating seals imperfections, creating a smooth, glossy finish that repels water and contaminants, minimizing maintenance. With added anti-scratch technology, Carbon Force enhances shine and provides long-lasting protection, making it a compelling choice for car enthusiasts [52].	Chemical Guy	4–5 years
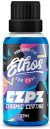 EZPZ Ceramic Coating	EZPZ Ceramic Polymer Coating offers a DIY-friendly approach to ceramic car coatings. Its Polymersilazane formula provides up to 3 years of hydrophobic protection, repelling water and contaminants for a cleaner, glossier finish. The flexible co-polymers allow for easier application and a longer work time, even in challenging conditions. Designed as a topcoat, EZPZ is compatible with other coatings and promises professional results without the hassle [53].	Ethos	2–3 years
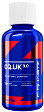 CQUK Ceramic Quartz Coating	With broad pH tolerance (3–14), and wide application temperature range (3–40°C/40F–100F) ensure robust protection against various environmental factors and ease of use. The formula’s high silica-quartz content creates a hard, protective layer, enhancing gloss and offering resistance to chemicals, salt, and UV damage. The simple wipe-on/wipe-off application further adds to its user-friendliness [54].	CARPRO™	1–2 years
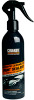 Rapid Ceramic Paint Sealant	Promises a stunning, easy-to-maintain finish, boasting exceptional gloss and shine, extreme hydrophobicity repelling water and dirt, and unsurpassed slickness. The coating simplifies washing and drying, while its application process is quick and easy, involving spraying and wiping. Coating’s durability, lasting for months due to its true inorganic ceramic technology [55].	CERAKOTE®	1–2 years
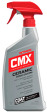 CMX Ceramic Spray Coating	Its simple spray-and-wipe application, suitable for both wet and dry surfaces, promises a showroom shine in minutes. The coating bonds to the paint, creating a protective layer that enhances shine and repels contaminants. While optimal results are achieved on clean, defect-free surfaces, the ease of application and potential for multiple coats make it an appealing option for car owners seeking convenient and effective ceramic protection [56].	Mothers® Polish	1–2 years
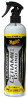 Ultimate Ceramic Coating	Meguiar’s Ultimate Ceramic Coating offers DIY-friendly, professional-grade ceramic protection for your car. This easy-to-apply spray coating enhances gloss, slickness, and water beading, while also concealing minor paint imperfections. Safe for various surfaces, including paint, trim, and PPF, it provides durable protection similar to professional ceramic coatings, but with a simplified application process. Proper surface preparation and maintenance are recommended for optimal results [57].	Meguiar’s	3–4 years
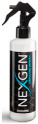 Ceramic Spray Silicon Dioxide	Offers a user-friendly approach to long-lasting car protection. Its graphene-infused formula promises up to a year of protection against UV rays, water spots, and contaminants, while enhancing gloss and depth. The spray-on application simplifies the process, eliminating the need for professional detailing expertise. The coating’s thermal properties allow for application in direct sunlight without streaking or spotting and also help prevent hard water spots. Safe for various surfaces like paint, glass, wheels, and trim [58].	Nexgen	1–2 years

**Table 4:** Different types of graphene coatings offered by different car manufacturers [72].

Product	Product Characteristics	Manufacturer	Average Paint Protection Time
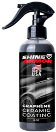 Spray type Graphene Ceramic Coating	Infuses graphene and ceramic to form a powerful coating that enhances vehicles protection, slickness, and durability. Powerful hydrophobic properties and high resistance to harmful UV rays, water, dirt, grimes, dust and debris. Can be used on all exterior vehicle surfaces such as wheel, glass, headlights, plastic, trim & etc. DIY friendly [73].	Shine Armor	6–12 months
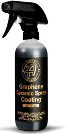 Graphene Ceramic Spray Coating	Infused with UV tracers that activate with UV blue light, coating become more visible in glossy looks under sunlight. Safe to use on painted surfaces, wheels, glass, headlights, plastic trim, bed liners, tonneau covers, canvas tops, floor mats, chrome and unfinished metal. DIY friendly [74].	Adam’s Polishes	18–20 months
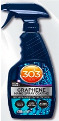 Graphene Nano Spray Coating	High resistance against UV rays, water spotting, fading & cracking. Has enhanced gloss finished properties. Formulated from graphene -oxide nano coating that can be applied on car’s paint, trim, & windows. DIY friendly [75].	303 Products	±12 months
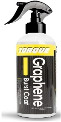 Graphene Burst Coat Coating	Repels any weather, chemical or roadside contaminants. Graphene Burst’s proprietary formula blends nano silica dioxide particles providing endless glass shine. Hydrophilic surface so water sheets off instead of beading. Improved resistance against stains & scratches. Has high contact angle and lower sliding angle; i.e., less stains, scratches & containments. DIY friendly [76].	Torque Detail	±12 months
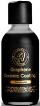 Advanced Graphene Ceramic Coating	A clear, nanocrystalline coating that protects vehicle from weather, chemicals, and UV rays while rejecting water, dirt and other deposits. 10H hardness and more than 7 years of protection. Has higher scratch and stain resistance features. Can be applied on paint, glass, headlights, chrome, wheels and trims. Used by professional car detailers [77].	Adam’s Polishes	84 months
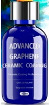 Advanced Graphene Coating	Updated version coating hardness increases to 12H so that it lasts longer. Superior resistance from salt fog corrosion, bird’s dropping, UV light, scratches, crushed stones and iron filings. Outstanding water beading that makes water almost jump off the surface. Used by professional car detailers. Used by professional car detailers [78].	Weiruixin	±120 months

## Data Availability

No new data were created or analyzed in this study.
